# Supplementary material for: Over-the-counter carrageenan-based sprays may interfere with PCR testing of nasopharyngeal swabs to detect SARS-CoV-2
Source: PLoS One. 2025 Feb 6;20(2):e0316700. doi: 10.1371/journal.pone.0316700 (PMC11801711; doi:10.1371/journal.pone.0316700)
Supplement: S1 Table — (PDF) [file pone.0316700.s001.pdf]

E-gene	Lower 95% CI of mean	Upper 95% CI of mean	Mean
Sample Only	25.1	25.17	25.14
Heparin Stock	N/A	N/A	N/A
Heparin 1/8	35.94	39.18	37.56
Heparin 1/32	30.39	33.85	32.12
CG Stock	N/A	N/A	N/A
CG 1/8	N/A	N/A	38.35
CG 1/32	27.28	28.73	28.01
Sample Only + Hz	24.66	25.36	25.01
Heparin Stock + Hz	25.83	26.58	26.21
Heparin 1/8 + Hz	25.03	25.53	25.28
Heparin 1/32 + Hz	25.01	25.5	25.25
CG Stock + Hz	N/A	N/A	N/A
CG 1/8 + Hz	N/A	N/A	38.38
CG 1/32 + Hz	27.08	28.31	27.70

S1. 95% Confidence Intervals (CI) for the Ct values of the E-gene from samples presented in Figure 3
