# Supplementary material for: Over-the-counter carrageenan-based sprays may interfere with PCR testing of nasopharyngeal swabs to detect SARS-CoV-2
Source: PLoS One. 2025 Feb 6;20(2):e0316700. doi: 10.1371/journal.pone.0316700 (PMC11801711; doi:10.1371/journal.pone.0316700)
Supplement: S2 Table — (PDF) [file pone.0316700.s002.pdf]

RdRP/S-gene	Lower 95% CI of mean	Upper 95% CI of mean	Mean
Sample Only	24.92	25.85	25.38
Heparin Stock	N/A	N/A	N/A
Heparin 1/8	36.85	41.96	39.41
Heparin 1/32	32.10	35.34	33.72
CG Stock	N/A	N/A	N/A
CG 1/8	35.99	40.13	38.06
CG 1/32	28.43	30.17	29.3
Sample Only + Hz	25.23	25.80	25.52
Heparin Stock + Hz	26.14	27.84	26.99
Heparin 1/8 + Hz	25.05	26.54	25.8
Heparin 1/32 + Hz	25.06	26.26	25.66
CG Stock + Hz	N/A	N/A	N/A
CG 1/8 + Hz	34.11	40.67	37.39
CG 1/32 + Hz	27.41	30.15	28.78

S2. 95% Confidence Intervals (CI) of the Ct values from the RdRP/S-gene of samples presented in Figure 3
