# Supplementary material for: Over-the-counter carrageenan-based sprays may interfere with PCR testing of nasopharyngeal swabs to detect SARS-CoV-2
Source: PLoS One. 2025 Feb 6;20(2):e0316700. doi: 10.1371/journal.pone.0316700 (PMC11801711; doi:10.1371/journal.pone.0316700)
Supplement: S3 Table — (PDF) [file pone.0316700.s003.pdf]

N-gene	Lower 95% CI of mean	Upper 95% CI of mean	Mean
Sample Only	24.95	25.25	25.10
Heparin Stock	N/A	N/A	N/A
Heparin 1/8	36.16	36.88	36.52
Heparin 1/32	32.43	33.32	32.87
CG Stock	N/A	N/A	N/A
CG 1/8	34.61	40.52	37.57
CG 1/32	28.87	30.47	29.67
Sample Only + Hz	24.73	25.76	25.24
Heparin Stock + Hz	26.42	27.10	26.76
Heparin 1/8 + Hz	25.28	25.78	25.53
Heparin 1/32 + Hz	25.20	25.97	25.59
CG Stock + Hz	N/A	N/A	N/A
CG 1/8 + Hz	31.07	43.79	37.43
CG 1/32 + Hz	28.13	29.10	28.62

S3. 95% Confidence Intervals (CI) of the N-gene Ct values from samples presented in Figure 3
