# Supplementary material for: Over-the-counter carrageenan-based sprays may interfere with PCR testing of nasopharyngeal swabs to detect SARS-CoV-2
Source: PLoS One. 2025 Feb 6;20(2):e0316700. doi: 10.1371/journal.pone.0316700 (PMC11801711; doi:10.1371/journal.pone.0316700)
Supplement: S4 Table — (PDF) [file pone.0316700.s004.pdf]

Internal Control	Lower 95% CI of mean	Upper 95% CI of mean	Mean
Sample Only	23.60	25.08	24.34
Heparin Stock	N/A	N/A	N/A
Heparin 1/8	35.25	38.24	36.74
Heparin 1/32	31.19	34.03	32.61
CG Stock	N/A	N/A	N/A
CG 1/8	35.79	39.11	37.45
CG 1/32	27.38	29.35	28.36
Sample Only + Hz	24.43	24.80	24.62
Heparin Stock + Hz	25.66	26.13	25.90
Heparin 1/8 + Hz	24.28	25.12	24.70
Heparin 1/32 + Hz	23.59	25.41	24.50
CG Stock + Hz	N/A	N/A	N/A
CG 1/8 + Hz	N/A	N/A	N/A
CG 1/32 + Hz	27.89	29.02	28.45

S4. 95% Confidence Intervals (CI) for the internal control of samples presented in Figure 3
