# Supplementary material for: Over-the-counter carrageenan-based sprays may interfere with PCR testing of nasopharyngeal swabs to detect SARS-CoV-2
Source: PLoS One. 2025 Feb 6;20(2):e0316700. doi: 10.1371/journal.pone.0316700 (PMC11801711; doi:10.1371/journal.pone.0316700)
Supplement: S5 Table — (PDF) [file pone.0316700.s005.pdf]

<table>
<tr><th>Study Day</th><th>Swab Type</th><th colspan="2">Ct Values Participant 1</th><th colspan="4">Ct Values Participant 2</th></tr>
<tr><td>Screening/Baseline</td><td>FLOQSwab</td><td>30.4 (i)</td><td>27.56 (r)</td><td colspan="4">26.50</td></tr>
<tr><td>Screening/Baseline</td><td>Rhinoswab</td><td>34.16 (i)</td><td>36.81 (r)</td><td>29.22 (i)</td><td colspan="2">29.82 (r)</td><td>32.55 (r)</td></tr>
<tr><td>Day 2</td><td>Rhinoswab</td><td>31.11 (i)</td><td>28.95 (r)</td><td colspan="4">Not collected</td></tr>
<tr><td>Day 3</td><td>FLOQSwab</td><td colspan="2">25.48</td><td colspan="4">24.77</td></tr>
<tr><td>Day 3</td><td>Rhinoswab</td><td colspan="2">25.04</td><td colspan="4">24.48</td></tr>
<tr><td>Day 5</td><td>FLOQSwab</td><td colspan="2">24.49</td><td colspan="4">25.33</td></tr>
<tr><td>Day 5</td><td>Rhinoswab</td><td colspan="2">25.09</td><td colspan="2">25.42 (i)</td><td colspan="2">24.65 ®</td></tr>
<tr><td>Day 10</td><td>FLOQSwab</td><td colspan="2">26.88</td><td colspan="4">25.42</td></tr>
<tr><td>Day 10</td><td>Rhinoswab</td><td colspan="2">26.3</td><td colspan="4">26.00</td></tr>
</table>

S5. Summary of IC Ct value results from samples which failed IC threshold test multiple times. (i) Initial testing, (r) repeat testing.
