# Supplementary material for: Over-the-counter carrageenan-based sprays may interfere with PCR testing of nasopharyngeal swabs to detect SARS-CoV-2
Source: PLoS One. 2025 Feb 6;20(2):e0316700. doi: 10.1371/journal.pone.0316700 (PMC11801711; doi:10.1371/journal.pone.0316700)
Supplement: Attachment — Submitted filename: PONE-D-24-31189 Highlighted.docx [file pone.0316700.s006.docx]

PLOS ONE

# Over-the-counter Carrageenan-based sprays may interfere with PCR testing of nasopharyngeal swabs to detect SARS-CoV-2

--Manuscript Draft--

| **Manuscript Number:** | PONE-D-24-31189 |
| --- | --- |
| **Article Type:** | Research Article |
| **Full Title:** | Over-the-counter Carrageenan-based sprays may interfere with PCR testing of nasopharyngeal swabs to detect SARS-CoV-2 |
| **Short Title:** | Carrageenan-mediated PCR interference of SARS-CoV-2 nasopharyngea lswabs |
| **Corresponding Author:** | Taylor Corocher Northern Health Epping, AUSTRALIA |
| **Keywords:** | SARS-CoV-2; Carrageenan; Heparin; glycosaminoglycan; INHERIT; Intranasal Heparin Trial; PCR Interference; Heparinase I |
| **Abstract:** | Carrageenan-containing nasal sprays, available over-the-counter (OTC), are often marketed as having anti-viral effects. Carrageenan belongs to the glycosaminoglycan family alongside heparin and has some structural similarities. Heparin is known to inhibit real-time quantitative polymerase chain reaction (RT-qPCR) in nasopharyngeal swabs used to detect SARS-CoV-2 and increase the occurrence of false negative results. We investigated of the interferent effect of carrageenan on RT-qPCR for SARS-CoV-2 detection on 4 different diagnostic platforms, demonstrating inaccurate and invalid results on the Seegene STARlet, whilst maintaining qualitative accuracy on the Cepheid GeneXpert, Roche Cobas LIAT, and Hologic Panther Aptima. Evidence of carrageenan interference on SARS-CoV-2 testing was consistent across two OTC brands and research-grade reconstituted iota-carrageenan, where 80% of samples for each carrageenan formulation produced invalid results. Further, a preliminary in vivo interference study demonstrated an increased Ct value within 15 minutes of dosage, with Ct values restored 60 minutes post-application. A direct comparison of carrageenan- and heparin-mediated PCR interference demonstrated that heparin interference is stronger at lower concentrations. Heparinase I treatment had no effect on carrageenan-interference. Taken together, this data suggests that carrageenan has an inhibitory effect on PCR that can result in false negative results, an effect which is currently not reversible, and could therefore have significant public health impacts on community testing of respiratory infectious diseases via PCR. |
| **Order of Authors:** | Taylor Corocher |
|  | Kira Edwards |
|  | Yvonne Hersusianto |
|  | Donald Campbell |
|  | Hui Yin Lim |
|  | Paul Monagle |
|  | Prahlad Ho |
| **Additional Information:** |  |
| **Question** | **Response** |
| **Financial Disclosure**  Enter a financial disclosure statement that describes the sources of funding for the work included in this submission. Review the [submission guidelines](http://journals.plos.org/plosone/s/submission-guidelines#loc-financial-disclosure-statement) for detailed requirements. View published research | Yes |

| articles from [*PLOS ONE*](https://journals.plos.org/plosone/) for specific examples.  This statement is required for submission and **will appear in the published article** if the submission is accepted. Please make sure it is accurate. |  |
| --- | --- |
| **Funded studies**  Enter a statement with the following details:   - Initials of the authors who received each award - Grant numbers awarded to each author - The full name of each funder - URL of each funder website - Did the sponsors or funders play any role in the study design, data collection and analysis, decision to publish, or preparation of the manuscript? |  |
| Did you receive funding for this work? |  |
| Please add funding details.  as follow-up to "**Financial Disclosure**  Enter a financial disclosure statement that describes the sources of funding for the work included in this submission. Review the [submission guidelines](http://journals.plos.org/plosone/s/submission-guidelines#loc-financial-disclosure-statement) for detailed requirements. View published research articles from [*PLOS ONE*](https://journals.plos.org/plosone/) for specific examples.  This statement is required for submission and **will appear in the published article** if the submission is accepted. Please make sure it is accurate. | Initials of the authors who received each award Paul Monagle, Donald Campbell  Grant numbers awarded to each author N/A  The full name of each funder  The Victorian Government Department of Jobs, Skills, Industry and Regions (DJSIR) URL of each funder website  Did the sponsors or funders play any role in the study design, data collection and analysis, decision to publish, or preparation of the manuscript?  No |

| **Funded studies**  Enter a statement with the following details:   - Initials of the authors who received each award - Grant numbers awarded to each author - The full name of each funder - URL of each funder website - Did the sponsors or funders play any role in the study design, data collection and analysis, decision to publish, or preparation of the manuscript? |  |
| --- | --- |
| Did you receive funding for this work?" |  |
| Please select the country of your main research funder (please select carefully as in some cases this is used in fee calculation).  as follow-up to "**Financial Disclosure**  Enter a financial disclosure statement that describes the sources of funding for the work included in this submission. Review the [submission guidelines](http://journals.plos.org/plosone/s/submission-guidelines#loc-financial-disclosure-statement) for detailed requirements. View published research articles from [*PLOS ONE*](https://journals.plos.org/plosone/) for specific examples.  This statement is required for submission and **will appear in the published article** if the submission is accepted. Please make sure it is accurate. | AUSTRALIA - AU |
| **Funded studies**  Enter a statement with the following details:   - Initials of the authors who received each award - Grant numbers awarded to each author - The full name of each funder - URL of each funder website - Did the sponsors or funders play any role in the study design, data collection and analysis, decision to publish, or preparation of the manuscript? |  |
| Did you receive funding for this work?" |  |
| **Competing Interests** | The authors have declared that no competing interests exist |

| Use the instructions below to enter a competing interest statement for this submission. On behalf of all authors, disclose any [competing interests](http://journals.plos.org/plosone/s/competing-interests) that could be perceived to bias this work—acknowledging all financial support and any other relevant financial or non- financial competing interests.  This statement is required for submission and **will appear in the published article** if the submission is accepted. Please make sure it is accurate and that any funding sources listed in your Funding Information later in the submission form are also declared in your Financial Disclosure statement.  View published research articles from  [*PLOS ONE*](http://journals.plos.org/plosone/) for specific examples. |  |
| --- | --- |
| **NO authors have competing interests**  Enter: *The authors have declared that no competing interests exist.*  **Authors with competing interests**  Enter competing interest details beginning with this statement:  *I have read the journal's policy and the authors of this manuscript have the following competing interests: [insert competing interests here]* |  |
| * typeset |  |
| **Ethics Statement**  Enter an ethics statement for this submission. This statement is required if the study involved:   - Human participants - Human specimens or tissue - Vertebrate animals or cephalopods - Vertebrate embryos or tissues | Alfred Hospital Human Research Ethics Committee (Project Number: 67614, NCT05204550)  Waiver of consent to use residual samples |

| - Field research   Write "N/A" if the submission does not require an ethics statement.  General guidance is provided below. Consult the [submission guidelines](http://journals.plos.org/plosone/s/submission-guidelines#loc-guidelines-for-specific-study-types) for detailed instructions. **Make sure that all information entered here is included in the Methods section of the manuscript.** |  |
| --- | --- |

| **Format for specific study types**  **Human Subject Research (involving human participants and/or tissue)**   - Give the name of the institutional review board or ethics committee that approved the study - Include the approval number and/or a statement indicating approval of this research - Indicate the form of consent obtained (written/oral) or the reason that consent was not obtained (e.g. the data were analyzed anonymously)   **Animal Research (involving vertebrate animals, embryos or tissues)**   - Provide the name of the Institutional Animal Care and Use Committee (IACUC) or other relevant ethics board that reviewed the study protocol, and indicate whether they approved this research or granted a formal waiver of ethical approval - Include an approval number if one was obtained - If the study involved *non-human primates*, add *additional details* about animal welfare and steps taken to ameliorate suffering - If anesthesia, euthanasia, or any kind of animal sacrifice is part of the study, include briefly which substances and/or methods were applied   **Field Research**  Include the following details if this study involves the collection of plant, animal, or other materials from a natural setting:   - Field permit number - Name of the institution or relevant body that granted permission |  |
| --- | --- |
|  |  |
| **Data Availability**  Authors are required to make all data underlying the findings described fully available, without restriction, and from the time of publication. PLOS allows rare exceptions to address legal and ethical concerns. See the [PLOS Data Policy](http://journals.plos.org/plosone/s/data-availability) and [FAQ](http://journals.plos.org/plosone/s/data-availability#loc-faqs-for-data-policy) for detailed information. | Yes - all data are fully available without restriction |

| A Data Availability Statement describing where the data can be found is required at submission. Your answers to this question constitute the Data Availability Statement and **will be published in the article**, if accepted. |  |
| --- | --- |
| **Important:** Stating ‘data available on request from the author’ is not sufficient. If your data are only available upon request, select ‘No’ for the first question and explain your exceptional situation in the text box. |  |
| Do the authors confirm that all data underlying the findings described in their manuscript are fully available without restriction? |  |
| **Describe where the data may be found in full sentences. If you are copying our sample text, replace any instances of XXX with the appropriate details.**   - If the data are **held or will be held in a public repository**, include URLs, accession numbers or DOIs. If this information will only be available after acceptance, indicate this by ticking the box below. For example: *All XXX files are available from the XXX database (accession number(s) XXX, XXX.*). - If the data are all contained **within the manuscript and/or Supporting Information files**, enter the following: *All relevant data are within the manuscript and its Supporting Information files.* - If neither of these applies but you are able to provide **details of access elsewhere**, with or without limitations, please do so. For example:   *Data cannot be shared publicly because of [XXX]. Data are available from the XXX Institutional Data Access / Ethics Committee (contact via XXX) for researchers who meet the criteria for access to confidential data.*  *The data underlying the results presented in the study are available from (include the name of the third party* | All relevant data are within the manuscript and its Supporting Information files |

| *and contact information or URL).*   - This text is appropriate if the data are owned by a third party and authors do not have permission to share the data.   * typeset |  |
| --- | --- |
| Additional data availability information: |  |

Manuscript	[Click here to](https://www2.cloud.editorialmanager.com/pone/download.aspx?id=37140625&guid=70e2f94d-55f3-46aa-9900-23af56419fc3&scheme=1)

[access/download;Manuscript;CG_Manuscript_PLOS_ONE_Adju](https://www2.cloud.editorialmanager.com/pone/download.aspx?id=37140625&guid=70e2f94d-55f3-46aa-9900-23af56419fc3&scheme=1)

# 1 Over-the-counter Carrageenan-based sprays may interfere with PCR

2 **testing of nasopharyngeal swabs to detect SARS-CoV-2**
3 Corocher T^1,2^, Edwards K^1,2^, Hersusianto Y^1,3^, Campbell D^4,8^, Lim HY^1,2^, Monagle P^5,6,9,10^, and Ho P^1,2,7^
4 ^1^Northern Pathology Victoria, Northern Health, Epping, VIC, Australia
5 2 NorthErn Clinical diagnostics and ThrombovAscular Research (NECTAR) Centre, Northern Health, Epping,
6 VIC, Australia
7 ^3^Infectious Diseases, Northern Health, Epping, VIC, Australia
8 ^4^Hospital without Walls, Northern Health, Epping, VIC, Australia
9 ^5^Department of Paediatrics, University of Melbourne, Melbourne, VIC, Australia
10 ^6^Murdoch Children’s Research Institute, Melbourne, VIC, Australia
11 ^7^Department of Medicine – Northern Health, University of Melbourne, Epping VIC Australia
12 ^8^Department of Medicine – Southern Clinical School, Monash University, Clayton
13 VIC Australia
14 ^9^Department of Haematology, Royal Children’s Hospital, Parkville VIC, Australia
15 ^10^Kids Cancer Centre, Sydney Children’s Hospital, Randwick, NSW, Australia
16 **Corresponding Author:** Taylor Corocher (taylor.corocher@nh.org.au)
17 **Keywords:** SARS-CoV-2, Carrageenan, Heparin, glycosaminoglycan, INHERIT, Intranasal Heparin Trial,
18 PCR Interference, Heparinase I
19 **Abbreviations:** carrageenan	(CG), over-the-counter	(OTC), glycosaminoglycan	(GAG), real-time
20 quantitative polymerase chain reaction (RT-qPCR), nasopharyngeal (NP), cycle threshold (Ct), herpes
21 simplex virus (HSV), human papillomavirus (HPV), varicella-zoster virus (VZV), Universal Transport Medium
22 (UTM), internal control (IC).
23 **Word Count:** 3829
24 **Author Contributions/CRediT statement: T Corocher**: Methodology, Data Curation, Formal Analysis,
25 Investigation, Writing – Original Draft, Writing – Review & Editing, Visualisation. **K Edwards**:
26 Conceptualization, Methodology, Data Curation, Formal Analysis, Investigation, Writing – Original Draft,
27 Writing – Review & Editing, Visualization, Project Administration. **Y Hersusianto**: Writing – Review &
28 Editing, Supervision. **D Campbell**: Conceptualization, Writing – Review & Editing, Supervision, Funding
29 Acquisition. **HY Lim:** Resources, Supervision. **P Monagle**: Conceptualization, Writing – Review & Editing,
30 Supervision, Funding Acquisition. **P Ho**: Conceptualization, Writing – Review & Editing, Supervision
31 **Funding Sources**: This work was funded by a grant from the Victorian Government Department of Health.
32 RhinoSwabs were kindly supplied by RhinoMed Australia. Neither funding source had any role in the study
33 design, conduct of the research, collection, analysis or interpretation of data, in the writing of the report, or
34 the decision to submit the article for publication.
35 **Competing Interests:** The authors declare that they have no known competing financial interests or
36 personal relationships that could have appeared to influence the work reported in this paper.

37	**ORCID Details:** T Corocher: 0000-0003-1127-0500, K Edwards: 0000-0002-8169-5187, Y Hersusianto:

38	n/a, D Campbell: 0009-0006-2639-7001, P Monagle: 0000-0002-3970-8984, P Ho: 0000-0003-1875-3927

# 39 Abstract

40 Carrageenan-containing nasal sprays, available over the counter (OTC), are often marketed as having anti-
41 viral effects. Carrageenan belongs to the glycosaminoglycan family alongside heparin and has some
42 structural similarities. Heparin is known to inhibit real-time quantitative polymerase chain reaction (RT-
43 qPCR) in nasopharyngeal swabs used to detect SARS-CoV-2 and increase the occurrence of false negative
44 results. We investigated of the interferent effect of carrageenan on RT-qPCR for SARS-CoV-2 detection on
45 4 different diagnostic platforms, demonstrating inaccurate and invalid results on the Seegene STARlet,
46 whilst maintaining qualitative accuracy on the Cepheid GeneXpert, Roche Cobas LIAT, and Hologic Panther
47 Aptima. Evidence of carrageenan interference on SARS-CoV-2 testing was consistent across two OTC
48 brands and research-grade reconstituted iota-carrageenan, where 80% of samples for each carrageenan
49 formulation produced invalid results. Further, a preliminary in vivo interference study demonstrated an
50 increased Ct value within 15 minutes of dosage, with Ct values restored 60 minutes post-application. A
51 direct comparison of carrageenan- and heparin-mediated PCR interference demonstrated that heparin
52 interference is stronger at lower concentrations. Heparinase I treatment had no effect on carrageenan-
53 interference. Taken together, this data suggests that carrageenan has an inhibitory effect on PCR that can
54 result in false negative results, an effect which is currently not reversible, and could therefore have significant
55 public health impacts on community testing of respiratory infectious diseases via PCR.

56

57 Abstract Word Count: 216

# 58 1. Introduction

59 One of the critical components in the successful management of the SARS-CoV-2 pandemic is the effective
60 and accurate PCR-testing for viruses. Our group is investigating use of a heparin-containing nasal spray as
61 a prophylactic against SARS-CoV-2 in the IntraNasal Heparin Trial (INHERIT) (NCT05204550). Our recent
62 work demonstrated that heparin causes interference in SARS-CoV-2 PCR assays, resulting in an increase
63 in invalid and false negative results when testing nasopharyngeal (NP) swabs immersed in heparin, the
64 interference can be prevented through treatment with the enzyme heparinase I [1].
65 Heparin is structurally similar to the algae-derived glycosaminoglycan (GAG) carrageenan (CG) [2], which
66 is available in multiple markets as an over the counter (OTC) nasal spray purporting to have antiviral effects.
67 The global CG market was valued at USD$871 million in 2022 with a predicted growth rate of 5.4% by 2030
68 [3]. Carrageenan has three most commonly available structures, Iota-, Kappa- and Lambda-CG. Studies of
69 Iota-CG have demonstrated antiviral activity against herpes simplex virus (HSV), human papillomavirus
70 (HPV), varicella-zoster virus (VZV), and human rhinoviruses *in vivo* and *in vitro* [2,4–6]. Iota-CG may act to
71 prevent viral binding to heparan sulfate proteoglycans in host tissues, inhibiting viral replication [7,8].
72 Whether CG interferes with polymerase chain reaction (PCR) is unknown, but the structural similarity to
73 heparin suggests it may have a similar effect. Therefore, evidence of CG-mediated PCR interference in NP
74 samples potentially have significant public health implications. This study examines CG-mediated PCR
75 interference on multiple SARS-CoV-2 diagnostic testing platforms, in comparison to heparin-mediated PCR
76 interference, and the impact of treatment with the heparinase I enzyme.

# 77 2. Methods

## 78 2.1. Diagnostic tools, patient samples and ethical approval

79 Northern Pathology Victoria (NPV), Australia, uses several platforms for SARS-CoV-2 detection: the
80 Seegene (In Vitro Diagnostics, Korea) STARlet liquid handling workstation in combination with the Seegene
81 Allplex SARS-CoV-2 assay (amplification of the SARS-CoV-2 E-gene, N-gene and RdRP/S-gene), plus
82 either the Seegene STARMag extraction kit, or the TANbead nucleic acid extraction kit in conjunction with
83 the MAELSTROM 9610 nucleic extractor (both Taiwan Advanced Nanotech inc, Taiwan), with PCR
84 amplification performed on the C1000 touch thermocycler (Bio-rad, USA). Other platforms used include the
85 Panther system with the Aptima SARS-CoV-2 Assay (Hologic, USA), the GeneXpert Xpress CoV-2 cartridge
86 (Cepheid, USA), and the Cobas LIAT with the Cobas SARS-CoV-2 and Influenza A/B Assay (Roche
87 Diagnostics, Switzerland).
88 Residual samples collected from SARS-CoV-2 positive patients were retained at NPV after routine clinical
89 testing; these samples were initially collected by NP swab sampling into 3 mL Copan Universal Transport
90 Medium (UTM; Copan Diagnostics, Italy), and stored at -80 °C after initial testing. A waiver of consent for
91 the use of residual samples for this research was granted by the Alfred Hospital Human Research Ethics
92 Committee (Project Number: 67614, NCT05204550). Samples used in the following experiments were

93	accessed between 06/02/2023 and 21/07/2023.

## 94 2.2. Carrageenan interference across diagnostic testing platforms

95 To investigate if CG-mediated PCR interference occurred, a pool of residual samples from SARS-CoV-2
96 positive patients (with an aggregate Ct value of 25) was prepared and separated into 3 mL aliquots. The
97 swab submersion method was performed as described in [1], using FLO Travel nasal spray (1.6 mg/mL CG,
98 consisting of 1.2 mg/mL iota-CG and 0.4 mg/mL Kappa-CG, Aspen Pharmacare, Australia, unless stated
99 otherwise). Each sample was tested using the assays described in section 2.1, with a dilution gradient of
100 FLO Travel nasal spray.

## 101 2.3. Carrageenan interference consistency across different formulations

102 In a second experiment, consistency of CG interference was investigated using two brands of commercially
103 available nasal sprays: (FLO Travel nasal spray 1.6 mg/mL and BETADINE Cold Defense nasal spray 1.2
104 mg/mL Iota-CG, iNova Pharmaceuticals, Singapore), and reconstituted Iota-CG powder (Sigma-Aldrich,
105 USA) in 0.9% NaCl (to 1.6 mg/mL). Ten SARS-CoV-2 positive samples were pooled (with an aggregate Ct
106 value of ~25 across genes tested) and then diluted to a total volume of 10 mL in UTM and split into three 3
107 mL aliquots, and the remaining 1 mL was set aside as the sample-only control. For each 3 mL aliquot, the
108 swab submersion method was performed with a 1/8 dilution of each CG formulation and tested on the
109 Seegene platform with the Allplex SARS-CoV-2 assay.
110 As the different brands of nasal sprays tested had different CG concentrations, to enable equal comparison
111 of the degree of interference between formulations, a series of dilutions was used. For clarity, the dilution
112 factor used will be referred to, rather than the heparin or CG concentrations.

## 113 2.4. A direct comparison of heparin and carrageenan PCR interference and the

114 **efficacy of heparinase I against carrageenan**
115 A comparison of heparin (5,000 IU/mL; Sigma Aldrich, USA) and carrageenan (FLO Travel nasal spray 1.6
116 mg/mL) was performed by testing a dilution series of each interferent on a pool of residual SARS-CoV-2
117 positive material. Nucleic acid was extracted on the Seegene STARlet using the STARMag extraction kit,
118 and extracted samples were then treated with 10 uL of 0.4 IU/mL heparinase I (IBEX technologies, Canada)
119 (incubated at room temperature for 30 minutes), and PCR setup was performed using the Seegene Allplex
120 SARS-CoV-2 assay on the C1000 touch thermal cycler. Samples were tested in triplicate and run in parallel
121 to avoid any confounding variables. To ensure the viability of heparinase I used, control samples were
122 prepared from pooled residual SARS-CoV-2 material and included in each assay run. These controls
123 contained 1) sample-only; 2) sample + spiked heparin; 3) sample + spiked heparin + heparinase I treatment.
124 Control 1 acts as the baseline for an inhibited reaction, control 2 demonstrates the increased Ct value due
125 to the addition of heparin, and control 3 is expected to return to within 2 Ct values of control 1 if heparinase
126 I is active.
127 To test the efficacy of heparinase I (IBEX technologies, Canada) against iota-CG, the swab submersion
128 method was performed as described in [1], using FLO Travel nasal spray. Nucleic acid extraction was
129 performed using the STARMag extraction kit on the Seegene STARlet. Subsequently, 10 uL of 0.4 IU/mL
130 heparinase I was added to extracted nucleic acid and incubated at room temperature for 30 minutes, before
131 PCR set up on the Seegene STARlet using the Allplex SARS-CoV-2 assay on the C1000 touch thermal
132 cycler. Heparinase I was reconstituted in a buffer made of 20 mM TRISMA base, 600 mM NaCl, and 150
133 mM CaCl2 adjusted to a pH of 7.0 with HCl.
134 **2.5. *In vivo* interference testing**
135 To determine the effects of CG used on SARS-CoV-2 testing *in vivo*, NP swabs were collected from an
136 author who was using FLO-travel nasal spray (KE). A baseline swab was collected prior to administration of
137 the nasal spray, swabs were then collected at 15 minutes, 30 minutes, 45 minutes, 60 minutes, 2 hours, 4
138 hours, 8 hours, 16 hours, and 24 hours post-dose; this was repeated on three separate occasions (biological
139 replicate, n=3). Each swab was collected in 2 mL of COPAN UTM (1 mL removed prior to collection) and
140 later spiked with 1 mL of pooled SARS-CoV-2 positive material. Different SARS-CoV-2 positive pools were
141 used to spike each set of biological replicates, samples were stored at -80°C and defrosted at RT prior to
142 analysis in technical triplicate using the STARMag extraction and Allplex SARS-CoV-2 assay kits, as per
143 manufacturers’ instructions.

## 144 2.6. Statistical Analysis

145 Ct values presented in this work are an average of three technical triplicates. For the *in vivo* interference
146 testing, the change in Ct value was normalized by subtracting the baseline sample Ct values from the Ct
147 values of each time point post-dose samples. Confidence intervals (CI) were calculated using descriptive
148 statistics in Prism (GraphPad, Dotmatics). On the Seegene platform, Ct values >40 cycles are considered
149 invalid, which is defined as a failure of the internal control (IC) to detect amplification of a known sequence
150 at a predetermined concentration.

# 151 3. Results

## 152 3.1. Analysis of carrageenan interference across diagnostic testing platforms

153 Pooled SARS-CoV-2 positive material was divided into aliquots and spiked with varying dilutions of FLO
154 Travel nasal spray, ranging from the stock concentration (1.6 mg/mL) to a 1/32 dilution. CG spiked samples
155 were then tested across different diagnostic platforms for any inhibitory effect as described in Sections 2.2

156	& 2.2).

157

158 **Figure 3-1: The effect of carrageenan on different diagnostic platforms: A pool of SARS-CoV-2 residual patient**
159 **material was divided into 3 mL aliquots. The swab submersion method was performed using a stock**
160 **concentration of carrageenan, a 1/2 dilution, 1/4 dilution, 1/8 dilution, 1/16 dilution, and a 1/32 dilution. Samples**
161 **were tested on the GeneXpert, the Roche Liat, the Hologic Panther, and the Seegene STARlet in combination**
162 **with the STARMag or TanBead extraction kits (as indicated). Mixed results were defined as differences in results**
163 **across the technical triplicates (eg. Negative/positive/one-gene positive etc)**
164 Where the CG concentration was greater than ¼ dilution, analysis with both the STARMag and Tanbead
165 extraction kits returned invalid results (**Error! Reference source not found.**). In contrast, the Hologic
166 Panther, GeneXpert, and LIAT platforms correctly detected SARS-CoV-2 genetic material at all
167 concentrations of CG tested (**Error! Reference source not found.**).
169 **Figure 3-2: The effect of CG interference on Ct values: A pool of SARS-CoV-2 was created from residual patient**
170 **samples and divided into 3 mL aliquots. CG was diluted from stock (1.6 mg/mL) to 1/32 of the recommended**
171 **dosage concentration. The swab submersion method was performed, and samples were tested on A) Cepheid**
172 **GeneXpert with the Xpress CoV-2/Flu/RSV cartridge, and B) using the STARMag extraction kit and Allplex**
173 **SARS-CoV-2 assay.**
174 However, correctly determining the presence or absence of SARS-CoV-2 in a sample does not confirm the
175 absence of CG-mediated PCR interference. Analysis of the Ct values from the GeneXpert (Figure 3-2A) and
176 Seegene STARMag assays (Figure 3-2B) demonstrate an increasing delay in Ct values with increasing CG
177 concentration, with Ct values increasing by ten when samples are spiked with undiluted CG solution (in
178 comparison to the sample-only control). No evidence of interference was seen at CG concentrations lower
179 than the 1/4 dilution. However, the Seegene STARlet appears to be more susceptible to the effects of PCR
180 interference at lower concentrations of CG with invalid results in the presence of undiluted CG.
181 Consistency of CG-mediated PCR interference across different formulations was examined by comparing
182 1/8 dilutions of FLO Travel nasal spray, Betadine spray, and CG reconstituted in 0.9% NaCl (Table 3-1).
183 Residual material from 10 patient samples was tested as described in Section 2.2.
184 The SARS-CoV-2 positive agreement for each formulation of CG compared to the ‘Sample-only’ control are
185 presented in Table 3-1, showing a consistent 20% positive agreement across tested formulations (Table

186	3-1).

|  | Sample-only  Control | Sigma  Carrageenan | FLO-travel | Betadine |
| --- | --- | --- | --- | --- |
| Positive | 10 | 2 | 2 | 2 |
| Invalid | 0 | 8 | 8 | 8 |
| Positive agreement % |  | 20% | 20% | 20% |

187 **Table 3-1 SARS-CoV-2 positive agreement of carrageenan brands (1/8 dilution), performed using the STARMag**
188 **extraction kit and the Allplex SARS-CoV-2 assay.**

## 189 3.2. Direct comparison of heparin and carrageenan interference on SARS-CoV-

190 **2 testing, and subsequent heparinase I treatment**
191 Finally, a comparison of the degree of heparin and CG interference was performed using pooled SARS-
192 CoV-2 positive material (as per Section 2.4). Tested concentrations of CG and heparin were the stock
193 concentration, 1/8, and 1/32 dilutions (Figure 3-3). Both heparin and CG, at stock and 1/8 dilutions, returned
194 either an invalid result or a positive result with Ct values approaching the cut-off threshold of 40 – a marked
195 increase when compared to the sample-only control Ct values of ~25 Ct. The 1/32 dilution of CG had an
196 internal control (IC) Ct of 28.36, vs the sample-only Ct of 24.34. In contrast, the 1/32 dilution of heparin had
197 an IC Ct of 32.61 (Supplementary Table 6-4). The trend of higher Ct values in the presence of heparin
198 compared to CG was similar across all genes tested (Supplementary Table 6-1, Table 6-2, Table 6-3).
199 After treatment with heparinase I, samples containing heparin resulted in Ct values comparable to the
200 sample-only control, while treated samples containing CG remained at higher Ct values.
202 **Figure 3-3: A comparison of PCR interference between heparin and carrageenan and the effect of heparinase I**
203 **treatment: A pool of residual SARS-CoV-2 material was split into 3 mL aliquots. For each aliquot, the swab**
204 **submersion method was used with either heparin (red bars) or CG (blue bars) at the following concentrations:**
205 **stock (1.6 mg/mL), 1/8 dilution, 1/32 dilution. For each dilution, 6 aliquots were prepared alongside a sample-**
206 **only control. Heparinase I was added to half the aliquots prepared after nucleic acid extraction (using the**
207 **STARMag extraction kit) and incubated at room temperature for 30 minutes. PCR setup was performed with**
208 **the Allplex SARS-CoV-2 assay and run on the thermocycler. (CG = Carrageenan, Hz = Heparinase I, IC = Internal**
209 **Control)**
210 **3.3. *In vivo* interference testing**
211 A pilot experiment was performed to determine if the interfering effect of CG would be seen *in vivo*, and to
212 examine how long after administration of a single dose of a CG-containing nasal spray this interference was
213 evident in resulting Ct values (Section 2.5). To assess changes in Ct value over time, post-dose sample Ct
214 values were normalized by subtraction of the baseline (0) time point Ct value (Figure 3-4). There was an
215 increase of ~3-4 Ct values across all three genes tested and the IC using the Allplex SARS-CoV-2 assay
216 on samples collected 15 minutes post-dose. This increase in Ct values was no longer evident at the 30
217 minutes post-dose timepoint.
219 **Figure 3-4: Determination of the presence of *in vivo* CG-mediated PCR inhibition: A baseline swab was collected**
220 **(time 0), followed by administration of BETADINE Cold Defense nasal spray. Subsequent swabs were collected**
221 **at 15 minutes, 30 minutes, 45 minutes, 60 minutes, 2 hours, 4 hours, 8 hours, 16 hours, and 24 hours post-dose.**
222 **Each swab was collected in 2 mL of UTM and spiked with 1 mL from pooled SARS-CoV-2 positive material.**
223 **Results shown are the mean of 3 biological replicates, normalized against the sample-only control, all samples**
224 **were run in three technical replicates. Data points represent the average of three biological replicates (n = 3).**
225 **(IC = Internal Control).**
226 Additional in vivo information comes from our clinical trial (INHERIT) examining the use of intranasal heparin
227 as a prophylactic to prevent transmission of SARS-CoV-2 within a household. Having defined the degree of
228 heparin-mediated PCR interference prior to commencement of the INHERIT study (5), strict protocols were
229 developed for the processing and analysis of samples collected from trial participants to ensure the accuracy
230 of trial data in the presence of a known PCR-interferent. Because our previous investigations showed that
231 in heparin-spiked samples Ct values post-heparinase I treatment are within 1-3 Ct values of their initial Ct
232 value when un-spiked, we set a threshold IC Ct value of <27 for trial runs (regardless of whether or not
233 SARS-CoV-2 is detected) as an indication of potential interferent presence, and as confirmation that the
234 heparinase I treatment has worked. Samples with ICs outside this range undergo repeat testing to satisfy
235 this requirement.
236 To date, the majority of participant samples analyzed pass this IC threshold test, with those that initially fail
237 passing upon repeat testing. However, our attention was drawn to a subset of samples from two participants,
238 from independent households, which consistently returned IC Ct values >27, despite repeat testing, and
239 with no other samples in those runs showing evidence of systemic PCR interference.
240 After investigation, it was discovered that both participants had been using a nasal spray containing CG
241 within 24 hours prior to consenting to be trial participation, and collection of the screening NP swabs by the
242 study nurse, and before starting treatment with the study drug. Results for both participants showed IC Ct
243 values >27 at screening, and in swabs collected on subsequent days after starting treatment the degree of
244 interference as seen in the Ct values decreased (Supplementary Table 6-5).
245 Two swab types are used for sample collection during the trial, the traditionally used FLOQ Swab and a two-
246 pronged Rhino swab. Generally, higher Ct values were seen with Rhino swab-collected samples. The
247 difference in degree of interference seen between sample collection methods could be due to the extended
248 time that the Rhino swab has contact with the nasal mucosa (~1 minute) when sampling, compared to the
249 FLOQ Swab (~10 seconds per nostril).

# 251 4. Discussion

## 252 4.1. The effect of carrageenan across SARS-CoV-2 diagnostic testing platforms

253 The data presented in **Error! Reference source not found.** demonstrates that CG-based nasal sprays,
254 which is readily available OTC and marketed as preventing or lessening the symptoms of respiratory
255 illness, interfere with PCR testing on a number of commercially available diagnostic testing platforms.
256 Qualitatively, we demonstrate that the presence of CG has an interfering effect (Error**! Reference source**
257 **not found.**) in samples tested, particularly affecting the STARMag extraction and Allplex SARS-CoV-2
258 assay kits. Similarly, despite not showing qualitative discrepancies in results, there was a significant
259 increase in CT values in the assays up to 10 cycles. A potential reason these qualitative results were
260 preserved may be the low starting CT value of our test COVID sample (25 compared to 32 in our previous
261 studies). Hence, the clinical impact of this interference is significant and could significantly impact results in
262 patients, and a larger impact would be seen in samples with higher starting Ct values, which is an indication
263 of a lower viral titer in the sample.
264 We have also noted a differential impact of the different assays. This may be in part due to the differential
265 impact of the underlying technology used. Panther, for example, appears to have minimal interference
266 impact from both carrageenan and heparin. One of the reasons hypothesized is the specificity of the
267 Panther’s magnetic beads which contain oligonucleotides complimentary to SARS-CoV-2 nucleic acid,
268 instead of relying only on the electromagnetic attraction of nucleic acid to the beads.
269 Similarly, when looking at the Ct values returned by the GeneXpert assay (Figure 3-2A), we can see an
270 increase when CG is present, indicating interference, however the degree of interference was not sufficient
271 to cause an invalid or false negative result. However, the comparative increase in Ct values between the
272 GeneXpert and See gene Allplex SARS-CoV-2 assays was much smaller (Figure 3-2B), indicating that the
273 Allplex assay may be more susceptible to interfering agents than the GeneXpert. This is consistent with us
274 data from the heparin study [1]. Unfortunately, due to the proprietary nature of diagnostic assays and
275 platforms, we were unable to determine if they had different technical characteristics that may have led to
276 these differences in results.
277 Furthermore, we also confirmed that this inhibitory PCR effect is seen across all different subtypes of
278 carrageenan, with all three formulations of CG at 1/8 dilution failed to detect SARS-CoV-2 in 8 out of 10
279 samples. This data suggest that it is a class effect of carrageenan, independent of any compounds used.
280 The difference described above, while small, may both have a significant impact in monitoring public health
281 measures and also affect results from clinical studies. There are a number of studies in the literature
282 investigating CG as potential antiviral treatment, where viral status is determined using PCR methodologies,
283 and thus, used to assess the efficacy of CG as an antiviral [9–14]. As our data shows that CG has an
284 interfering effect on PCR that artificially elevates Ct values, this suggests the results of these studies should
285 be interpreted with a degree of caution, as findings of higher rates of negative test results or higher Ct values
286 post-treatment could be due to the impact of CG interference on PCR efficiency, and not necessarily an
287 indication of treatment efficacy. Likewise, studies that do not account for differences in PCR efficiency, such
288 as presenting the IC Ct values, or where results are not normalized against an endogenous control of known
289 concentration, should also be interpreted with caution.

## 290 4.2. A comparison of heparin and carrageenan PCR interference

291 When directly comparing carrageenan and heparin interference on the same sample material, carrageenan
292 had a lower interference impact, and its interferent effect was only noted at higher concentrations (Figure
293 3-3). Part of the reason for this may be the minor structural differences seen between heparin and
294 carrageenan. For example, Heparin is a highly negatively charged biomolecule, with a density charge of
295 3.3[15], so it is likely to outcompete nucleic acids when binding to magnetic beads, However Iota-CG has a
296 density charge of only 1.53 [16], which suggests it would be less successful in displacement of nucleic acids
297 when binding to magnetic beads, which could explain lower degree of interference seen in comparison to
298 heparin.
299 Given the structural similarities with heparin, we initially hypothesized that heparinase may be able to reverse
300 the interference. Unfortunately, heparinase I treatment of CG-containing samples showed little change in Ct
301 values compared to the untreated CG-containing samples (Figure 3-3), thus ruling it out as a method of
302 removing interference. Further work is required to find a reliable method to remove carrageenan interference
303 in the laboratory.

## 304 4.1. *In vivo* carrageenan interference analysis

305 To further test the carrageenan interference impact in the real-world setting, we explored in-vivo the effect
306 of carrageenan spray over a 24-hour period (Figure 3-4). There was a clear increase in Ct values at 15
307 minutes post-dose, with Ct values returning close to baseline levels at 30 minutes post-dose. A change in
308 IC Ct values of up to 1 is expected due standard variance within a run, so an increase of 3-5 Ct values
309 between baseline and 15 minutes post-dose (when tested on the same run) suggests that there is little CG
310 remaining in the nasal passages after 30 minutes post-dose. With the limitations of this experiment being
311 performed on a single individual, it suggests that any NP swabs intended for diagnostic PCR testing should
312 be collected a minimum of 30 minutes post-dose when administering intranasal CG, as a precaution.
313 Whether prolonged use leads to extension of the time of interference through achievement of a steady state
314 is unknown.
315 The presence of PCR interference for up to two days after CG dosage as seen in trial participants suggests
316 that the data shown in Figure 3 4 may be an under-representation of the ability of CG remaining in the nasal
317 passages post-dosage to cause PCR interference. This is not surprising, as the subject of the in vivo
318 experiment only administered one dose, and had not used the product consistently over a period of time,
319 whereas the trial participants indicated that they had been using the product multiple times per day over an
320 extended period.
321 While more data is required to make a definitive statement, the above examples provide ‘real-world’
322 evidence that CG-mediated PCR interference can occur outside of an experimental laboratory context, and
323 suggests that there may be definitive consequences when diagnostic PCR testing is performed on NP swabs
324 collected from people using CG containing nasal sprays.

## 325 4.2. Limitations

326 The use of laboratory spiked samples creates limitations when assessing PCR interference, as the amount
327 and length of time that CG that remains in the nasal passage after administration is unknown, and the
328 concentrations tested here may be under- or over-representative compared to what would be found in
329 samples from people using intranasal sprays containing CG. However, the examples above do provide
330 confidence that our experimental results are also be applicable in the real world. The *in vivo* pilot study to
331 determine how long after administration of CG-based nasal sprays is an n of 1, and must be further
332 investigated before definitive conclusions can be made.

## 333 4.3. Conclusions

334 We demonstrate here that CG has an inhibitory impact on RT-qPCR, similar to that seen in the presence of
335 heparin (Figures 3.1 – 3.4). To our knowledge, this is the first study to demonstrate CG-mediated PCR
336 interference, and does raise the impact of these sprays on future public health responses, as well as studies
337 using carrageenan and PCR assays. In conjunction with the *in vivo* experiment demonstrating absence of
338 PCR interference after 30 minutes post-drug administration, the use of any nasal products should be
339 considered when looking at the validity of any clinical result. Further clinical studies are required to validate
340 these findings.

## 341 4.4. Acknowledgements

342 We would like to acknowledge Caitlin McQueen Thomson for proof-reading the article.

# 343 5. References

344 1.	Edwards K, Corocher T, Hersusianto Y, Campbell D, Subbarao K, Neil JA, et al. Heparin-mediated
345 PCR interference in SARS-CoV-2 assays and subsequent reversal with heparinase I. J Virol 346	Methods. 2024; 114944. doi:10.1016/J.JVIROMET.2024.114944
347 2.	Necas	J,	Bartosikova	L.	Carrageenan:	a	review.
348 https://vetmed.agriculturejournals.cz/doi/1017221/6758-VETMED.html.	2013;58:	187–205. 349	doi:10.17221/6758-VETMED
350 3.	Carrageenan Market Size & Share Analysis Report, 2030. [cited 1 Feb 2024]. Available:
351 https://[www.grandviewresearch.com/industry-analysis/carrageenan-market](http://www.grandviewresearch.com/industry-analysis/carrageenan-market)
352 4.	Laurie C, El-Zein M, Franco EL, Coutlée F. Assessment of the possible inhibitory effect of
353 carrageenan in human papillomavirus DNA testing by polymerase chain reaction amplification. J 354	Med Virol. 2021;93: 6408–6411. doi:10.1002/JMV.26963
355 5.	Mahomoodally MF, Lobine D, Rengasamy KRR, Gowrishankar S, Tewari D, Zengin G, et al. Marine
356 Algae: A Potential Resource of Anti-HSV Molecules. Processes 2019, Vol 7, Page 887. 2019;7: 887. 357	doi:10.3390/PR7120887

358	6.	Pangestuti R, Kim SK. Biological activities of carrageenan. Adv Food Nutr Res. 2014;72: 113–124.

359	doi:10.1016/B978-0-12-800269-8.00007-5

360 7.	Shi Q, Wang A, Lu Z, Qin C, Hu J, Yin J. Overview on the antiviral activities and mechanisms of
361 marine	polysaccharides	from	seaweeds.	Carbohydr	Res.	2017;453–454:	1–9. 362	doi:10.1016/J.CARRES.2017.10.020

363	8.	Laurienzo P. Marine Polysaccharides in Pharmaceutical Applications: An Overview. Mar Drugs. 364	2010;8: 2435. doi:10.3390/MD8092435

365 9.	Eccles R, Meier C, Jawad M, Weinmüllner R, Grassauer A, Prieschl-Grassauer E. Efficacy and safety
366 of an antiviral Iota-Carrageenan nasal spray: a randomized, double-blind, placebo-controlled
367 exploratory study in volunteers with early symptoms of the common cold. 2010 [cited 14 Aug 2023]. 368	doi:10.1186/1465-9921-11-108
369 10.	Eccles R, Winther B, Johnston SL, Robinson P, Trampisch M, Koelsch S. Efficacy and safety of iota-
370 carrageenan nasal spray versus placebo in early treatment of the common cold in adults: the ICICC 371	trial. 2012. doi:10.1186/s12931-015-0281-8
372 11.	Fazekas T, Eickhoff P, Pruckner N, Vollnhofer G, Fischmeister G, Diakos C, et al. Lessons learned
373 from a double-blind randomised placebo-controlled study with a iota-carrageenan nasal spray as
374 medical device in children with acute symptoms of common cold. BMC Complement Altern Med. 375	2012;12: 1. doi:10.1186/1472-6882-12-147
376 12.	Ludwig M, Enzenhofer E, Schneider S, Rauch M, Bodenteich A, Neumann K, et al. Efficacy of a
377 Carrageenan nasal spray in patients with common cold: a randomized controlled trial. Respir Res. 378	2013;14: 1. doi:10.1186/1465-9921-14-124
379 13.	Bovard D, van der Toorn M, Schlage WK, Constant S, Renggli K, Peitsch MC, et al. Iota-carrageenan
380 extracted from red algae is a potent inhibitor of SARS‐CoV-2 infection in reconstituted human airway 381	epithelia. Biochem Biophys Rep. 2022;29: 101187. doi:10.1016/J.BBREP.2021.101187
382 14.	Fröba M, Große M, Setz C, Rauch P, Auth J, Spanaus L, et al. Iota-Carrageenan Inhibits Replication
383 of SARS-CoV-2 and the Respective Variants of Concern Alpha, Beta, Gamma and Delta. 2021 [cited 384	14 Aug 2023]. doi:10.3390/ijms222413202
385 15.	Weiss RJ, Esko JD, Tor Y, Biomol O, Author C. Targeting Heparin-and Heparan Sulfate-Protein
386 Interactions HHS Public Access Author manuscript. Org Biomol Chem. 2017;15: 5656–5668. 387	doi:10.1039/c7ob01058c
388 16.	Hugerth A, Sundelöf LO. Effect of polyelectrolyte counterion specificity on dextran sulfate-
389 amphiliphile interaction in water and aqueous/organic solvent mixtures. Langmuir. 2000;16: 4940– 390	4945. doi:10.1021/LA9916880

391

392

# 393 6. Supplementary

| **E-gene** | **Lower 95% CI of mean** | **Upper 95% CI of mean** | **Mean** |
| --- | --- | --- | --- |
| Sample Only | 25.1 | 25.17 | 25.14 |
| Heparin Stock | N/A | N/A | N/A |
| Heparin 1/8 | 35.94 | 39.18 | 37.56 |
| Heparin 1/32 | 30.39 | 33.85 | 32.12 |
| CG Stock | N/A | N/A | N/A |
| CG 1/8 | N/A | N/A | 38.35 |
| CG 1/32 | 27.28 | 28.73 | 28.01 |
| Sample Only + Hz | 24.66 | 25.36 | 25.01 |
| Heparin Stock + Hz | 25.83 | 26.58 | 26.21 |
| Heparin 1/8 + Hz | 25.03 | 25.53 | 25.28 |
| Heparin 1/32 + Hz | 25.01 | 25.5 | 25.25 |
| CG Stock + Hz | N/A | N/A | N/A |
| CG 1/8 + Hz | N/A | N/A | 38.38 |
| CG 1/32 + Hz | 27.08 | 28.31 | 27.70 |

394 **Table 6-1: 95% Confidence Intervals (CI) for the Ct values of the E-gene from samples presented in Figure 3.3;**
395 **Results are presented as the mean of three technical replicates. N/A denotes samples that returned invalid**
396 **results; CG = carrageenan, Hz = heparinase I.**

| **RdRP/S-gene** | **Lower 95% CI of mean** | **Upper 95% CI of mean** | **Mean** |
| --- | --- | --- | --- |
| Sample Only | 24.92 | 25.85 | 25.38 |
| Heparin Stock | N/A | N/A | N/A |
| Heparin 1/8 | 36.85 | 41.96 | 39.41 |
| Heparin 1/32 | 32.10 | 35.34 | 33.72 |
| CG Stock | N/A | N/A | N/A |
| CG 1/8 | 35.99 | 40.13 | 38.06 |
| CG 1/32 | 28.43 | 30.17 | 29.3 |
| Sample Only + Hz | 25.23 | 25.80 | 25.52 |
| Heparin Stock + Hz | 26.14 | 27.84 | 26.99 |
| Heparin 1/8 + Hz | 25.05 | 26.54 | 25.8 |
| Heparin 1/32 + Hz | 25.06 | 26.26 | 25.66 |
| CG Stock + Hz | N/A | N/A | N/A |
| CG 1/8 + Hz | 34.11 | 40.67 | 37.39 |
| CG 1/32 + Hz | 27.41 | 30.15 | 28.78 |

397 **Table 6-2: 95% Confidence Intervals (CI) of the Ct values from the RdRP/S-gene of samples presented in Figure**
398 **3.3; Results are presented as the mean of three technical replicates. N/A denotes samples that returned invalid**
399 **results; CG = carrageenan, Hz = heparinase.**

| **N-gene** | **Lower 95% CI of mean** | **Upper 95% CI of mean** | **Mean** |
| --- | --- | --- | --- |
| Sample Only | 24.95 | 25.25 | 25.10 |
| Heparin Stock | N/A | N/A | N/A |
| Heparin 1/8 | 36.16 | 36.88 | 36.52 |
| Heparin 1/32 | 32.43 | 33.32 | 32.87 |
| CG Stock | N/A | N/A | N/A |
| CG 1/8 | 34.61 | 40.52 | 37.57 |
| CG 1/32 | 28.87 | 30.47 | 29.67 |
| Sample Only + Hz | 24.73 | 25.76 | 25.24 |
| Heparin Stock + Hz | 26.42 | 27.10 | 26.76 |
| Heparin 1/8 + Hz | 25.28 | 25.78 | 25.53 |
| Heparin 1/32 + Hz | 25.20 | 25.97 | 25.59 |
| CG Stock + Hz | N/A | N/A | N/A |
| CG 1/8 + Hz | 31.07 | 43.79 | 37.43 |
| CG 1/32 + Hz | 28.13 | 29.10 | 28.62 |

400 **Table 6-3 95% Confidence Intervals (CI) of the N-gene Ct values from samples presented in Figure 3.3, Results**
401 **are presented as a mean of three technical replicates. N/A is present where samples returned invalid results,**
402 **CG = carrageenan, Hz = heparinase.**

| **Internal Control** | **Lower 95% CI of mean** | **Upper 95% CI of mean** | **Mean** |
| --- | --- | --- | --- |
| Sample Only | 23.60 | 25.08 | 24.34 |
| Heparin Stock | N/A | N/A | N/A |
| Heparin 1/8 | 35.25 | 38.24 | 36.74 |
| Heparin 1/32 | 31.19 | 34.03 | 32.61 |
| CG Stock | N/A | N/A | N/A |
| CG 1/8 | 35.79 | 39.11 | 37.45 |
| CG 1/32 | 27.38 | 29.35 | 28.36 |
| Sample Only + Hz | 24.43 | 24.80 | 24.62 |
| Heparin Stock + Hz | 25.66 | 26.13 | 25.90 |
| Heparin 1/8 + Hz | 24.28 | 25.12 | 24.70 |
| Heparin 1/32 + Hz | 23.59 | 25.41 | 24.50 |
| CG Stock + Hz | N/A | N/A | N/A |
| CG 1/8 + Hz | N/A | N/A | N/A |
| CG 1/32 + Hz | 27.89 | 29.02 | 28.45 |

404 **Table 6-4: 95% Confidence Intervals (CI) for the internal control of samples presented in Figure 3.3, Results are**
405 **presented as a mean of three technical replicates. N/A is present where samples returned invalid results, CG =**
406 **carrageenan Hz = heparinase.**

| **Study Day** | **Swab Type** | **Ct Values Participant 1** | | **Ct Values Participant 2** | | | |
| --- | --- | --- | --- | --- | --- | --- | --- |
| Screening/Baseline | FLOQSwab | 30.4 (i) | 27.56 (r) | 26.50 | | | |
| Screening/Baseline | Rhinoswab | 34.16 (i) | 36.81 (r) | 29.22 (i) | 29.82 (r) | | 32.55 (r) |
| Day 2 | Rhinoswab | 31.11 (i) | 28.95 (r) | Not collected | | | |
| Day 3 | FLOQSwab | 25.48 | | 24.77 | | | |
| Day 3 | Rhinoswab | 25.04 | | 24.48 | | | |
| Day 5 | FLOQSwab | 24.49 | | 25.33 | | | |
| Day 5 | Rhinoswab | 25.09 | | 25.42 (i) | | 24.65 ® | |
| Day 10 | FLOQSwab | 26.88 | | 25.42 | | | |
| Day 10 | Rhinoswab | 26.3 | | 26.00 | | | |

407 **Table 6-5 Summary of IC Ct value results from samples which failed IC threshold test multiple times. (i) Initial**
408 **testing, (r) repeat testing.**

Figure 3-1	[Click here to access/download;Figure;Figure 3-1.tif](https://www2.cloud.editorialmanager.com/pone/download.aspx?id=37036460&guid=3f9be175-1a92-40cb-bf36-b2ee3dfe1c41&scheme=1)

Figure 3-2	[Click here to access/download;Figure;Figure 3-2.tif](https://www2.cloud.editorialmanager.com/pone/download.aspx?id=37036461&guid=3f21b1f9-6011-4cf6-8929-39d7ebbfc208&scheme=1)

Figure 3-3	[Click here to access/download;Figure;Figure 3-3.tif](https://www2.cloud.editorialmanager.com/pone/download.aspx?id=37036462&guid=1a1d9f96-bd55-4c2e-9a9c-e2b7b35cb2f6&scheme=1)

Figure 3-4	[Click here to access/download;Figure;Figure 3-4.tif](https://www2.cloud.editorialmanager.com/pone/download.aspx?id=37036463&guid=97461479-b737-4b15-968b-edd671912ffa&scheme=1)
